# The study of movement skills in sports: toward an integrative approach

**DOI:** 10.3389/fpsyg.2023.1252201

**Published:** 2023-10-27

**Authors:** Sigmund Loland, Gertjan Ettema, Øyvind Sandbakk

**Affiliations:** ^1^Norwegian School of Sport Sciences, Oslo, Norway; ^2^Norwegian University of Science and Technology, Trondheim, Norway

**Keywords:** movement skills, mechanistic explanations, phenomenology, integrative approach, sports

## Abstract

The article commences with a fundamental objective: to comprehend movement skills in sports in a manner that can bridge the dualist gap between experiential qualities observed in practice and theoretical and mechanistic explanations. Drawing inspiration from Kuhn’s concept of scientific paradigms, practical examples from skiing research, and innovative insights into the integration of phenomenology and mechanistic explanation in cognitive science, we have outlined a three-step integrative approach. The first step entails the development of phenomenological descriptions of the primary experiential qualities inherent in the execution of the skills being investigated. In the second step, phenomenological descriptions play a pivotal role by setting constraints and delineating a space for the elaboration of multilevel mechanistic analyses. These analyses draw upon insights from various fields, encompassing biomechanics, motor control approaches, expertise studies, and cognitive science. The third step involves the systematization of findings and the formulation of sport-specific movement skills theories. We contend that such theories hold substantial significance as they serve as valuable supplements to skill studies conducted within rigid, nomological frameworks. Sport-specific theories include descriptions of first-person experiential qualities and can contribute to bridging the theory-practice gap effectively.

## Introduction

Human movement skills, understood as functional and efficient solutions to movement tasks, can be studied in several ways. One main approach is based on natural science in which the body and its movements are described in objective, quantitative terms and explained mechanistically with the help of scientific disciplines such as biomechanics and motor control and learning. For example, a traditional motor control approach starts from insights into the neuromuscular interplay between a hierarchy of central nervous system (CNS) structures and sensory inputs from the involved structures (e.g., muscles). Skill development is explained by the plasticity and adaptability of the human organism.

A second approach emphasizes the subjective first-person perspective and analyses experiential qualities in movement. With inspiration from phenomenology, descriptions of movement skill execution are presented with concepts such as rhythm and flow and with references to specific experiential qualities as found in the ‘tribal’ language of athletes and coaches. The approach can connect to constructivist approaches in the social sciences, where learned movement patterns are seen as outcomes of socialization within the context of cultural norms and values.

There are major differences between these approaches. They are based on different epistemological and methodological premises. The mechanistic approach offers explanations and predictions within well-defined theoretical frameworks. The phenomenological approach represents the quest to describe and understand practice as ‘lived’ and experienced by practitioners.

The question to be addressed here is whether the mechanistic and phenomenological approaches are mutually exclusive or whether the apparent gaps can be overcome in scientifically sound ways. Firstly, and with the help of Kuhn’s idea of scientific paradigms, we will discuss the challenge of paradigmatic incommensurability, questioning to what extent and in what sense such a challenge may exist between phenomenological and mechanistic accounts. Secondly, and with examples from existing sports research, we will examine limitations and possibilities in the handing this challenge. With backing in works by Montero (2016) and Pokropski (2021) in particular, we will point to solutions in which phenomenology and mechanistic approaches can be integrated. Thirdly, and on this basis, we will outline the main steps of an integrative approach to movement skills.

## Kuhn, paradigms, and incommensurability

Kuhn’s (1962/1996) work on the structure of scientific revolutions, particularly his conceptualization of scientific paradigms, has exerted a significant impact on the understanding of scientific development. Although receiving criticism for his theory of paradigm change (Mizrahi, 2018), the scientific paradigm concept has proven fertile and has become a standard reference in academic discourse. In the most extensive interpretation, a scientific paradigm consists of a disciplinary matrix, that is, a basic pattern of metaphysical assumptions, theoretical and methodological concepts, and best practice guidance that, for some time, is a commonly accepted framework within a scientific discipline or research area (Bird, 2022).

For example, a biomechanical study of movement skills from a third-person point of view offers quantified descriptions, mechanistic explanations, and predictive force. The alternative phenomenological approach examines the experiential qualities of movement skills. The methodological ideal is to describe the world as lived *(Lebenswelt)* and explore skill execution from a first-person point of view.

In Kuhnian terminology, the two approaches could be conceived as incommensurable and belong to different paradigmatic traditions (Oberheim and Hoyningen-Huene, 2018; Bird, 2022). One indication of incommensurability is that the main theoretical and methodological concepts of one approach can not be translated and applied in meaningful ways in another (Boyd, 1991). For example, references to mechanical forces in movement are not commonly used in a description of experiential qualities, and phenomenological analyses referring to experiential qualities do not make sense in a biomechanical analysis.

This is not necessarily problematic. The original Kuhnian idea of scientific revolutions, in which the hegemonic scientific paradigm is challenged and eventually replaced with a new and incommensurable paradigm, is contested. As Toulmin (1970) and later Argamakova (2018) argue, empirically speaking, few, if any, scientific breakthroughs can be explained in this way. Current grand projects in the life sciences and neuroscience build on interdisciplinary and transdisciplinary multi-paradigmatic approaches. The alternative view is that different scientific paradigms are not mutually exclusive but complementary. For example, a combination of approaches to movement skills can enhance insight and understanding of the phenomenon.

Still, there is a challenge here. What emerges as a unified phenomenon in real life – an athlete performing at a high level of skill where all movement elements are integrated into efficient wholes – is split into several explanatory and interpretative schemes. Although research necessarily builds on the reduction of complexity, separating bodies of knowledge can prevent the integration of ideas and data and thereby limiting our understanding. On the other hand, if separate bodies of knowledge are bridged and integrated, their complexity may enrich our understanding.

Historically, the natural science and phenomenological schisma reflect classic Cartesian dualism in which the body and mind belong to two separate and qualitatively different spheres of reality: extended, material, and mechanistic substance *(res extensa)*, and non-extended non-mechanistic thinking substance *(res cogitans).* In contemporary science and philosophy of mind, dualism has lost the ground it once possessed, yet the mind–body explanatory gap remains in numerous different versions, some of which are of special relevance in this context. Let us illustrate with examples from skiing research.

## Current research on movement skills: limitations and possibilities

Consider the complex movement skill in the sport of ski jumping. Athletes experience their sport as a ‘contest against gravity.’ A peak experience of a successful jump is the feeling of ‘flying,’ ‘not landing,’ most clearly obtained in the largest hills where athletes appear to take off again after literally having scraped the surface.

Typically, practitioners’ accounts of embodied, experiential qualities play a marginal role in research. The standard approach is biomechanics. Core elements are the role of gravity, ground reaction forces, including the centripetal component in the curvature of the inrun, force development in the take-off action, and aerodynamics. From this perspective, athletes do not ‘fly’ but predominantly fall and glide, postponing the landing, which is possible due to the smart use of aerodynamic forces.

The reductionism of the approach, that is, the attempt to describe and explain a phenomenon in an analytic breakdown into its basic entities, limits knowledge outcomes. Anecdotally, a simulation study on the mechanics of the inrun action in ski jumping revealed a complex mathematical problem at the end of the radius when entering the straight take-off table of the ski jump (Ettema et al., 2005). In the moments before take-off, the centripetal ground reaction force component and rotation abruptly disappear. In the real world, the well-trained ski jumper has no issue with this change in condition. However, solving this issue mathematically and simulating the real world appeared difficult.

The next step of the study was to add perspectives from neural control and imperfection.^1^ The ski jumping simulation indicated that the rate of muscle force generation needed to manage the transitions between radius of the middle part of the inrun and the straight ski jump while maintaining body position was beyond feasible. Nevertheless, somehow motion must occur. In real life, then, instructions like ‘maintain a constant position’ can become a quest for an ‘undesired Utopia’. The question arises as to whether small movements by the athlete during the inrun are an imperfection, a necessity, irrelevant, or perhaps even an advantage.^2^ In other words, the mechanistic approach (biomechanics) seems to miss the essential qualities of the phenomenon it aspires to explain: good ski jumping technique.^3^

Another inadequacy is the lack of conceptualization of movement skill innovation and development. The quest for performance enhancement is a core aspect of an athlete’s skill training. An example from cross-country skiing is illustrative. In the early 2000s, the Swedish skier Bjørn Lind sprinted faster than any other skier in the double poling technique. A skier’s movements and speed can be explained by analyzing the frequency of double poling along with cycle lengths. Lind could produce longer cycle lengths than other skiers by using the legs extensively in a forward ‘jump-like movement’ in the repositioning phase before he dropped his body mass on the poles in the repositioning phase. This was later described as the ‘modern’ double poling technique (Holmberg et al., 2005). The underlying mechanics were further investigated 10 years later. The active and rapid extension of the legs to rise and forward-rotate the center of mass increased the potential and rotational energy, in which an effective energy transfer to mechanical energy and propulsion through the poles in the subsequent poling phase would enable fast double poling speeds (Danielsen et al., 2015).

Mechanistic analyses are efficient in the critical assessment of already existing movement skills. The focus is primarily retrospective in kind. In a more comprehensive approach, insights must be integrated from actual practice in which movement creativity and innovation play important parts.

## Interplay: toward a non-reductive physicalist point of view

The quest for a broader, integrative approach faces paradigmatic tensions. This concerns not only theoretical and methodological issues but metaphysical assumptions as well, among them views on the body–mind relationship and the nature of consciousness. A detailed discussion of various positions is beyond the scope of this essay. A brief review, however, is necessary to understand the premises of our further argument.

Basically, we face here what Chalmers (1995) calls ‘the hard problem of consciousness’: how to explain the relationship between physical phenomena, such as the neuromuscular interplay between sensory inputs in the execution of movement skills, and the subjective experience of the same execution, or ‘what it is like’ from a first-person point of view.^4^

Several solutions have been proposed. To a reductive physicalist, mind is matter and can be explained in mechanistic terms. For instance, if we, sometime in the future, acquire sufficient insight into the neurophysiology and biochemistry of the brain, the subjective experience of skill execution can be fully explained by chains of cause-and-effect relationships. The hard problem of consciousness does not really exist.

The position is criticized on many accounts, among them that one and the same mental state, for instance, the discomfort of anaerobic fatigue during intensive training, can have a variety of realizers of both physiological and psychological kind. To explain a particular mental state with reference to one main explanatory scheme seems impossible. More generally, reductive physicalism is limited when it comes to accounts of the extensive variability and diversity in human interpretation and sense-making. Highly motivated athletes see anaerobic fatigue as a sign of high-quality training and development. ‘No pain, no gain’, as the slogan goes. Others react negatively and associate fatigue not with progress but with destructive pain.

To the classical Cartesian dualist, the solution is to see mind and matter as ontologically belonging to different spheres of reality. In modern terms: Neurophysiological response and its subjective interpretation have no relation. This naturally lends itself to a dualistic epistemology, where we operate in two different worlds understood by two different paradigms – one mechanistic and one interpretive. Whilst Cartesian dualism is no longer a viable ontology in research, the latter epistemological assumptions are common in the sports sciences (Loland and McNamee, 2017). For instance, the main body of research on movement skills is anchored in a mechanistic paradigm. In a comprehensive understanding of movement skills, the exclusivity of that focus is contra-intuitive. How can intentional movement be understood if the first-person perspective is ignored?

Contemporary cognitive science is dominated by a non-reductive physicalist point of view in which mental states have their origins in physical phenomena but cannot be fully explained in simple, mechanistic terms (Changeux, 1997, 2004). Varela et al.’s (2016) study of ‘the embodied mind’ interprets consciousness by integrating phenomenological (and Buddhist) accounts of human experience with cognitive science. Varela et al. point to the position of ‘enactivism’: The living body is seen as a self-producing and self-maintaining system that enacts and brings forward relevance and meaning in the world. Embodied cognition, for instance in the execution of movement skills, finds its form in complex, senso-motoric interaction with the world.

Gallagher and Zahavi (2012) refers to the 4 E’s of an enactivist approach: Cognition is not a matter of inner representations of events in the world but is embodied (anchored in senso-motoric and perceptual capabilities of the body), embedded (intentional and always directed toward objects and events of the world), enacted (focusing on affordable objects and events), and extended (emerging in a deep interaction with environmental objects and events).

As will be shown below, the recent attempt by Pokropski (2021) of phenomenological analysis as a starting point for an integration with multi-level mechanistic explanation in cognitive science provides an interesting framework for movement skill studies. In the most extensive understanding, a particular experiential quality is the tip of the iceberg of an immensely complex interplay between an individual’s biology and ‘lived’ history from the moment of conception to the moment of performance.

What has been said so far on the need for a broader, integrative approach is in line with a non-reductive physicalist position. Even if mechanistic and phenomenological approaches differ in theoretical and methodological frameworks, they are seen as complementary ways of describing the same physical reality. However, although being broader than what is found in traditional movement analysis, our scope is not the complete mapping of all contributing factors in skill development but the far more restricted aim of examining factors with immediate and significant impact in the execution of skills.

In the last decades, non-reductive physicalist ideas have exerted impact in movement skill studies. Newell’s (1986) work on the functional aspects of movement and the significance of interactions between organismic, environmental, and task constraints in coordination development has provided essential theoretical groundwork. Examples can be found in the works of Ettema and colleagues (Ettema et al., 2017, 2018, 2023; Løkkeborg and Ettema, 2020) on the impact of environmental or task constraints, such as incline, speed, and power demand, on the selection of sub-techniques in classical roller skiing. Still, there remain unanswered questions when it comes to integrating a first-person perspective. While their findings closely aligned with expectations based on the metabolic efficiency of these techniques, none of these factors could be identified as the sole controlling environmental parameter. Moreover, significant inter-individual differences were observed.

The phenomenon of hysteresis, as seen in the speed-dependent walk-run transition, was also evident in these studies. Hysteresis cannot be adequately explained solely by the concept of ‘minimizing energy expenditure’ but is better elucidated by dynamical systems models (e.g., Haken et al., 1985; Hommel et al., 2001). In this context, the ideas proposed by Wolpert et al. (1995), introducing the subjective comparison between intended, anticipated, and perceived results of movement, are particularly relevant. If hysteresis, essentially a delay in action, is a natural occurrence in sub-technique selection and disappears under artificial conditions (e.g., changing environmental/task constraints but not power demand), one could argue that the comparison between intended and anticipated outcomes is unnaturally influenced. It is worth noting, however, that these dynamical systems models do not seem to encompass the holistic phenomenological experience of athletes, including sensations described as ‘being in rhythm’ or ‘in the flow.’

A final example from cross-country skiing research includes an attempt to integrate the subjective, first-person point of view. Cross-country skiing technique has been analyzed by advanced global navigation satellite systems and inertial movement unit analyses combined with heart rate measurements and video (Tjønnås et al., 2019; Seeberg et al., 2022). The objective measurements allow the determination of the speed and position of the skier, cycle rate and length, body, ski, and poling angles, the timing of the movements, and indications of the metabolic effort. Thereafter experiential qualities can be captured in a more inclusive way by the athlete subjectively describing the race and positioning, the technical solutions, and the feeling of fatigue toward the finish line. Many experiential qualities might be well correlated with the objective data, demonstrating links to mechanistic explanations. Others might deviate and challenge both explanations and the experience of the skier. But again, some (important) experiential qualities, such as those of ‘flow’ or ‘rhythm’, may not be captured. There is need for alternative approaches.

## Executive knowledge: ‘knowing how’ versus ‘knowing that’

From the non-reductive physicalist perspective, the gap between the first and third-person point of view is primarily epistemological in kind. Ryle’s (1949/2009) distinction between ‘knowing-that’ and ‘knowing-how’ exemplifies this. ‘Knowing-that’ refers to being able to *describe and explain* a skill or competence, as in the biomechanics of successful skiing technique. ‘Knowing-how’ refers to the executive practical knowledge of *performing* the same skills as in actual well-performed skiing. Ryle criticizes what he sees as a dualist, ‘intellectualist legend’ with the implicit understanding of an ontologically independent mind, or a ‘ghost in the machine’, that secures successful outcomes by strict rule-following. Ryle’s point is the opposite: Execution of skills precedes and is independent of its articulated explanation.

In athlete and coach communities, the primary interest is in ‘knowing how’ expressed in vague and generic ‘tribal’ terms and in instructional nudges such as ‘finding the rhythm,’ ‘going all in,’ ‘being alert yet relaxed’, and ‘becoming one with the task’. What more can be said of ‘knowing how’?

One commonly held view emphasizes automatization. At their best, skill experts perform ‘without thinking’. Beilock and Carr (2001. p. 702) talk of the ‘expertise-induced amnesia’ hypothesis. The hypothesis finds some empirical support in Csikszentmihalyi’s (2008) theory of expert ‘flow’-experiences with an optimal tension between challenge and mastery and in Dreyfus et al. (1986) phenomenological-based theory of non-conceptual and smooth coping. The view is usually accompanied by the idea that if performers think critically while performing, skills break down into fragments, and performance quality decreases.^5^

In the philosophy of sport, this view has met significant critique (Moe, 2005; Breivik, 2014; Ilundáin-Agurruza, 2014; Borge, 2015; Montero, 2016; Birch, 2017). On a broader scale, Montero (2016) develops the critique and defends the alternative ‘cognition-in-action’ principle. Skill execution takes deliberate effort and includes critical problem-solving and self-teaching. Successful performances in fields such as dance, music, chess, and sports are characterized by clear performer intention, balanced effort, ongoing critical review and adjustment, and a quest for control. Negative and unpleasant thoughts are ‘washed away’.

Recent insights into the functioning of the brain provide empirical support to skill execution as various modes of deliberate and cognitive action. In their review of current research, and pointing to the work of, among others, J.-P. Changeux and G. W. Edelman, Farisco et al. (2017) portray the brain as a complex, dynamic, and plastic organ that is spontaneously active and predisposed to be projective in the evaluation and modeling of the world. Brain architecture opens for ‘… a complex flow of feedforward and feedback loops’ in explicit and aware as well as in implicit and unaware functional modes (Farisco et al., 2017, p. 2015).

Borge’s (2015, p. 125) distinctions between three modes of knowing how to do a sport seem to follow this line of reasoning. There is reflective awareness of knowing-how (as in Montero’s ‘cognition in action’), in-zone awareness of knowing-how (as in Csikszhentmihalyi’s *flow*), and even zoned-out awareness of knowing-how (as during the transportation stretches of a skiing race in which the racers are in control without focusing on the execution of their skiing technique). Bergamin (2017) and Christensen et al. (2016) portray a view of skilled action in which these modes of skill execution are ‘meshed’ and overlap and interact:

Automation has clear benefits for skill control: the integration and simplification of action control can make action production more efficient. But cognitive control nevertheless makes a vital contribution to skill control by determining the nature of the situation and configuring and adjusting lower-order sensorimotor processes appropriately. Cognitive and automatic processes thus characteristically operate together in an intimately meshed arrangement, with cognitive control typically focused on strategic task features and automatic control responsible for implementation.

The reference to sensorimotor processes indicates that this ‘meshed arrangement’ is closely connected with the proprioceptive sense.

## Proprioception and experiential qualities

In Montero’s (2016) view, *proprioception,* sometimes used synonymously with kinaesthesia, or the kinesthetic sense plays a core role. In Han et al.’s (2016) definition, the proprioceptive sense is ‘…an individual’s *ability* to integrate the sensory signals from mechanoreceptors to thereby determine body segment positions and movements in space’. More generally, the proprioceptive sense is defined as part of the somatosensory system and enables stability, accuracy, and efficiency in the solving of movement tasks by combining multiple inputs to the CNS from muscle and connective tissue receptors, as well as information from the vestibular system and the exteroceptive senses: vision, sound, and touch.

Proprioception is defined and explained not just physiologically but psychologically and contextually with impact from personality, sex, age, and situational factors such as the intensity and stress of a competitive situation. Montero (2016, p. 121) extends this perspective even further. In her view, proprioception is not just ‘…the sense by which we acquire information about the positions and movements of our bodies’, it is also ‘…an aesthetic sense, that is, a sense by means of which we experience beauty, grace, and other aesthetic properties’. Proprioception includes the phenomenological grasping of conceptualizable aesthetic experiences.

It should be noted that what is of interest is not the individual athlete’s subjective experience of a movement skill but the experiential, proprioceptive qualities that characterize the successful execution of the skill itself, its invariant and demarcating qualities in practice, so to speak. Montero’s core example is dance, where the many and various techniques have strict, intersubjective, and detailed prescriptions and where expressive, aesthetic elements play a key role. Examples from skiing can be the feel of perfect timing of the take-off in ski jumping or the sense of optimal power utilization in a double-poling sprint. The important point is that experiential qualities are representational: they are intersubjective qualities of lived practice that can be articulated and examined critically.

Insights into the experiential skill qualities are found not only among performers but in experienced observers as well. Discussing competent dance critics, Montero (2016, p. 201) talks of their reference to ‘kinaesthetic sympathy.’ In a similar vein, experiential qualities of sports skills are matters of shared understanding of both athletes and coaches and probably a key to their successful interaction.^6^

Whereas the practitioners’ language in dance deals with aesthetic qualities, ‘tribal’ coach and athlete language is influenced by biomechanics. In his study of alpine skiing technique, Loland (1992, 2008) has explored possible interconnections between experiential qualities and mechanistic explanations. Terms such as ‘being in balance,’ ‘finding support on the surface,’ and ‘optimal gliding’ can be operationalized in a series of detailed prescriptions on the use of hip and knee angling, and adaptive ski edging. Loland argues that most references to experiential qualities have their biomechanistic equivalents and can be translated into a mechanistic framework, thus suggesting complementarity. Balance deals with equilibrium conditions, and support from the surface and gliding with the efficient utilization and optimization of frictional forces. As with the dynamical systems approach, however, the integrating element, phenomenologically referred to as the pre-reflective and holistic sense of movement rhythm in which the execution of skills is experienced as a functional whole, seems to evade analytic operationalization. What can be done?

## Toward an integrative approach

Pokropski’ (2021) work on integrating phenomenology with cognitive science offers possibilities. Phenomenology is used to give an initial description of the phenomenon under study and provide constraints for exploring relevant mechanistic explanations. We take inspiration in Pokropski’s approach in our outline three steps of integrative approach to movement skills.

*The first step* is to articulate and tentatively decompose the experiential qualities of good technique. To be able to grasp essential qualities, expert movement ought to be studied *in situ* and with reference to practitioners’ sense and understanding. There is a need for a phenomenological methodology to describe the experts’ life world (*Lebenswelt*): the directly perceived, pre-theoretical experience of executing movement skills. This is the first-person perspective of the sensorily engaged ‘lived body’ operating in a perceptual field of numerous actual and potential interconnections (Merleau-Ponty, 2012). Montero’s (2016) descriptions of proprioception in dance, and Loland’s (2008) the experiential qualities of alpine skiing, are inspired by phenomenological descriptions of this kind.

With this first step, functional and dynamic constraints can be identified, creating a space for the search for further analyses and mechanistic explanations (Pokropski, 2021, 139 ff.). *Functional constraints* are found by decomposing the skill under study into its constitutive elements or defining its ‘functional architecture’, so to speak. For example, in Loland’s analysis, the experience of well-executed alpine skiing technique is decomposed into three main technical elements: being in balance, finding support on the surface, and smooth and effortless gliding. In our interpretation here, *dynamical constraints* refer to how these qualities emerge and play out temporally in skill execution. Phenomenologically, Loland refers to the holistic experiential quality of movement rhythm or movement flow.

The functional and dynamic constraints provide critical criteria in the search for possible mechanistic explanations. We are now at *the second step* of the integrative approach. The aim is not to integrate phenomenology into a well-defined nomological framework as found in classical biomechanics but to explore a patchwork of multilevel explanations from multiple research fields (Pokropski, 2021, 79 ff.). For instance, being in balance can be explained with reference to biomechanical analysis of dynamic stability and/or motor control approaches based on dynamical systems theory. Finding support on the surface and smooth gliding implies the efficient use of frictional forces. Again, biomechanics and motor control approaches are of relevance. In addition, expert skiers’ fine-tuned sense for gliding and optimizing frictional forces require additional insights from, say, the neuroscience of proprioception. Movement rhythm understood phenomenologically refers to a holistic whole that is bigger than the sum of its parts. Relevant explanations can be found in cognitive science and in insights into the brain’s spontaneous and adaptive capabilities in evaluating and modeling the world ‘in action’, and from expertise studies discussed in detail in Montero’s (2016).

The integration of experiential qualities and mechanistic explanations is a critical and explorative exercise. The attempt is to describe and explain ‘lived’ skill execution. As is evident from the ski jumping simulation study discussed above (Ettema et al., 2005), there is no hierarchical ordering here in which ‘incomplete’ phenomenology is converted into ‘complete’ scientific analysis. Phenomenological accounts of a skill can also lead to the revision of empirical hypotheses and choice of explanations. The integrative process is holistic in nature.

*A third step* implies using the outcome of step two in the systematic theory building of skill patterns. This is an exercise of connecting and bridging key concepts and explanations in complementary and consistent ways. A systematic overview of the phenomenological structure of a skill and its multilevel mechanistic explanations constitute a theory of this skill. Integrative approaches give rise to *specific skill theories*. With their starting point in experiential qualities of actual skill execution and the explorative approach into multi-level relevant mechanisms, specific skill theories are different from and can complement theories developed within stricter nomological structures. An integrative approach connects the first-person ‘lived’ perspective with the third-person perspective of mechanistic science.

## Concluding comments

We started this article by describing the need for a broader and integrated understanding of movement skills in sports that potentially bridges the gap between accounts of experiential qualities in practice and theoretical explanation. By starting from Kuhn’s idea of paradigms, using practical cases from skiing research and approaches as those found in among others Montero and Pokropski, we have sketched three steps of an integrative approach. Moreover, we have argued that the integrative approach can lead to sport-specific movement skill theories that complement traditional nomological movement science and strengthen the practical relevance of research.

Our outline is no ‘quick fix’ solution to bridging the theory-practice gap. Pokropski’s (2021) account of integrating phenomenology with cognitive science needs critical review and development (Ward, 2022; Madary, 2023). The very idea of integration is contested by critics who define phenomenology as a philosophical, transcendental perspective. Moreover, traditionally, scientific explanation is anchored in precise and conceptually clear theoretical frameworks. Attempts on explaining a skill with a pluralistic system of multilevel mechanistic explanations may seem challenging. No doubt, the integrative approach would require open-minded and multidisciplinary research efforts, substantial data processing power, and the exercise of a core scientific virtue: the non-reductive reduction of complexity.

The rewards might be worth the effort, however. Kuhn (1962/1996) argues that innovative insights and paradigmatic change often originate and develop at the margins of established scientific milieus. With its relatively short history and practical orientation, the sports sciences are at these margins. Further research along the lines of the integrative approach may lead to more innovative ways of understanding human skill execution. Expanding the perspective, the study of movement skills might be well-suited to shed new light on far more extensive questions, such as the nature of body–mind interaction and the nature of human consciousness.

## Author contributions

SL drafted and wrote the article with contributions from GE and ØS.

## Footnotes

^1^“No movement in sports is done perfectly; there is always room for improvement” is a popular expression. We may expand on this, positing that it does not need to be perfect to be beautiful, and neither does the control of movement; it simply needs to work.

^2^Defining ‘imperfection’ from a mechanistic perspective is doomed to be limited. Some movement is required to handle any perturbation in the inrun (including the abovementioned transitions), which is a positive factor. On the other hand, movement may increase aerodynamic drag, which is a negative factor. Moreover, movement may affect (control of) the take-off action, which is an undecided factor. To put it differently, when searching for optimization, scientists (Ettema et al., 2005) may interpret the practical challenge (proceeding through the inrun with high speed and in a position that allows a good jump) incorrectly, thereby ‘barking up the wrong tree’.

^3^Certainly, more complex mathematics that would have been able to solve the problem more elegantly exists, but such challenges arise most frequently in computer simulation and modeling. The use of integration solvers in a direct dynamics computer simulation is an example of not applying pure mathematics (algebra) when describing reality but searching for an optimal solution. Possibly, the development of artificial intelligence (AI) is the pinnacle of this ‘crusade’ and can provide new possibilities in this respect.

^4^See Nagel (1974) who argues that the subjective character of ‘what it is like’ to have a certain type of experience, for instance of well-ececuted technique, escapes physical theory.

^5^Anecdotally, and according to expert support staff, about half of Norwegian elite ski jumpers have no recollection of the take-off action right after the performance (having landed).

^6^There is a connection here to Rizzolatti and collaborators’ hypothesis in neuroscience of ‘the human mirror system’ – or of an in-built ‘action-observation network’ (Birch, 2017). When observing motor action, specialized mirror neurons in the brain exhibit increased activity. Research in monkeys relates to basic motor action: grasping, touching, *et cetera*. It can be hypothesized that similar activation takes place in humans and that the mirroring function plays a role in the learning and critical evaluation of movement skills. This provides further empirical evidence for a non-dualist approach to understanding skill execution (Birch, 2017) and may have interesting practical implications, among them as a rationale for imitation as a main didactic approach in skill learning (Watanabe et al., 2017).
